# Supplementary material for: Artificial Intelligence-Enhanced Precision Medicine Reveals Prognostic Impact of TGF-Beta Pathway Alterations in FOLFOX-Treated Early-Onset Colorectal Cancer Among Disproportionately Affected Populations
Source: Int J Mol Sci. 2025 Sep 17;26(18):9067. doi: 10.3390/ijms26189067 (PMC12469963; doi:10.3390/ijms26189067)
Supplement: Supplementary file 1 [file ijms-26-09067-s001.zip › ijms-3839315-supplementary.pdf]

Supplementary Figures and Tables:

Table S1. Comparison of Early-Onset Hispanic/Latino (H/L) Patients Treated with FOLFOX versus Not Treated with FOLFOX

TGF-beta Pathway			
Gene	Early-Onset Hispanic/Latino Treated with FOLFOX n (%)	Early-Onset Hispanic/Latino Not Treated with FOLFOX n (%)	p-value
**BMPR1A Mutation**			
Present	4 (5.5%)	2 (3.8%)	1
Absent	69 (94.5%)	50 (96.2%)	
**SMAD2 Mutation**			
Present	4 (5.5%)	3 (5.8%)	1
Absent	69 (94.5%)	49 (94.2%)	
**SMAD3 Mutation**			
Present	3 (4.1%)	3 (5.8%)	0.6923
Absent	70 (95.9%)	49 (94.2%)	
**SMAD4 Mutation**			
Present	9 (12.3%)	7 (13.5%)	1
Absent	64 (87.7%)	45 (86.5%)	
**TGFBR1 Mutation**			
Present	2 (2.7%)	2 (3.8%)	1
Absent	71 (97.3%)	50 (96.2%)	
**TGFBR2 Mutation**			
Present	3 (4.1%)	6 (11.5%)	0.1617
Absent	70 (95.9%)	46 (88.5%)	

Table S2. Comparison of Late-Onset Hispanic/Latino (H/L) Patients Treated with FOLFOX versus Not Treated with FOLFOX

TGF-beta Pathway			
Gene	Late-Onset Hispanic/Latino Treated with FOLFOX n (%)	Late-Onset Hispanic/Latino Not Treated with FOLFOX n (%)	p-value
**BMPR1A Mutation**			
Present	0 (0.0%)	0 (0.0%)	1
Absent	91 (100.0%)	50 (100.0%)	
**SMAD2 Mutation**			
Present	3 (3.3%)	4 (8.0%)	0.245
Absent	88 (96.7%)	46 (92.0%)	
**SMAD3 Mutation**			
Present	4 (4.4%)	4 (8.0%)	0.4541
Absent	87 (95.6%)	46 (92.0%)	
**SMAD4 Mutation**			
Present	16 (17.6%)	9 (18.0%)	1
Absent	75 (82.4%)	41 (82.0%)	
**TGFBR1 Mutation**			
Present	3 (3.3%)	1 (2.0%)	1
Absent	88 (96.7%)	49 (98.0%)	
**TGFBR2 Mutation**			
Present	5 (5.5%)	3 (6.0%)	1
Absent	86 (94.5%)	47 (94.0%)	

Table S3. Comparison of Early-Onset Non-Hispanic White (NHW) Patients Treated with FOLFOX versus Not Treated with FOLFOX

TGF-beta Pathway			
**Gene**	**Early-Onset NHW Treated with FOLFOX n (%)**	**Early-Onset NHW Not Treated with FOLFOX n (%)**	**p-value**
**BMPR1A Mutation**			
Present	4 (1.1%)	10 (3.3%)	0.05575
Absent	371 (98.9%)	292 (96.7%)	
**SMAD2 Mutation**			
Present	19 (5.1%)	16 (5.3%)	1
Absent	356 (94.9%)	286 (94.7%)	
**SMAD3 Mutation**			
Present	14 (3.7%)	12 (4.0%)	1
Absent	361 (96.3%)	290 (96.0%)	
**SMAD4 Mutation**			
Present	52 (13.9%)	37 (12.3%)	0.6144
Absent	323 (86.1%)	265 (87.7%)	
**TGFBR1 Mutation**			
Present	8 (2.1%)	9 (3.0%)	0.6506
Absent	367 (97.9%)	293 (97.0%)	
**TGFBR2 Mutation**			
Present	11 (2.9%)	16 (5.3%)	0.1721
Absent	364 (97.1%)	286 (94.7%)	

Table S4. Comparison of Late-Onset Non-Hispanic White (NHW) Patients Treated with FOLFOX versus Not Treated with FOLFOX

TGF-beta Pathway			
**Gene**	**Late-Onset NHW Treated with FOLFOX n (%)**	**Late-Onset NHW Not Treated with FOLFOX n (%)**	**p-value**
**BMPR1A Mutation**			
Present	15 (1.6%)	15 (2.3%)	0.4458
Absent	904 (98.4%)	638 (97.7%)	
**SMAD2 Mutation**			
Present	35 (3.8%)	43 (6.6%)	**0.0173**
Absent	884 (96.2%)	610 (93.4%)	
**SMAD3 Mutation**			
Present	29 (3.2%)	34 (5.2%)	0.05578
Absent	890 (96.8%)	619 (94.8%)	
**SMAD4 Mutation**			
Present	125 (13.6%)	104 (15.9%)	0.2244
Absent	794 (86.4%)	549 (84.1%)	
**TGFBR1 Mutation**			
Present	13 (1.4%)	12 (1.8%)	0.6482
Absent	906 (98.6%)	641 (98.2%)	
**TGFBR2 Mutation**			
Present	38 (4.1%)	46 (7.0%)	**0.01578**
Absent	881 (95.9%)	607 (93.0%)	

Table S5. Comparison of Early-Onset versus Late-Onset Hispanic/Latino (H/L) Patients Treated with FOLFOX

TGF-beta Pathway			
Gene	Early-Onset Hispanic/Latino Treated with FOLFOX n (%)	Late-Onset Hispanic/Latino Treated with FOLFOX n (%)	p-value
**BMPR1A Mutation**			
Present	4 (5.5%)	0 (0.0%)	**0.03747**
Absent	69 (94.5%)	91 (100.0%)	
**SMAD2 Mutation**			
Present	4 (5.5%)	3 (3.3%)	0.7011
Absent	69 (94.5%)	88 (96.7%)	
**SMAD3 Mutation**			
Present	3 (4.1%)	4 (4.4%)	1
Absent	70 (95.9%)	87 (95.6%)	
**SMAD4 Mutation**			
Present	9 (12.3%)	16 (17.6%)	0.4767
Absent	64 (87.7%)	75 (82.4%)	
**TGFBR1 Mutation**			
Present	2 (2.7%)	3 (3.3%)	1
Absent	71 (97.3%)	88 (96.7%)	
**TGFBR2 Mutation**			
Present	3 (4.1%)	5 (5.5%)	0.7334
Absent	70 (95.9%)	86 (94.5%)	

Table S6. Comparison of Early-Onset versus Late-Onset Hispanic/Latino (H/L) Patients Not Treated with FOLFOX

TGF-beta Pathway			
Gene	Early-Onset Hispanic/Latino Not Treated with FOLFOX n (%)	Late-Onset Hispanic/Latino Not Treated with FOLFOX n (%)	p-value
**BMPR1A Mutation**			
Present	2 (3.8%)	0 (0.0%)	0.4952
Absent	50 (96.2%)	50 (100.0%)	
**SMAD2 Mutation**			
Present	3 (5.8%)	4 (8.0%)	0.7127
Absent	49 (94.2%)	46 (92.0%)	
**SMAD3 Mutation**			
Present	3 (5.8%)	4 (8.0%)	0.7147
Absent	49 (94.2%)	46 (92.0%)	
**SMAD4 Mutation**			
Present	7 (13.5%)	9 (18.0%)	0.7205
Absent	45 (86.5%)	41 (82.0%)	
**TGFBR1 Mutation**			
Present	2 (3.8%)	1 (2.0%)	1
Absent	50 (96.2%)	49 (98.0%)	
**TGFBR2 Mutation**			
Present	6 (11.5%)	3 (6.0%)	0.4882
Absent	46 (88.5%)	47 (94.0%)	

Table S7. Comparison of Early-Onset versus Late-Onset Non-Hispanic White (NHW) Patients Treated with FOLFOX

TGF-beta Pathway			
**Gene**	**Early-Onset NHW Treated with FOLFOX n (%)**	**Late-Onset NHW Treated with FOLFOX n (%)**	**p-value**
**BMPR1A Mutation**			
Present	4 (1.1%)	15 (1.6%)	0.612
Absent	371 (98.9%)	904 (98.4%)	
**SMAD2 Mutation**			
Present	19 (5.1%)	35 (3.8%)	0.3824
Absent	356 (94.9%)	884 (96.2%)	
**SMAD3 Mutation**			
Present	14 (3.7%)	29 (3.2%)	0.7225
Absent	361 (96.3%)	890 (96.8%)	
**SMAD4 Mutation**			
Present	52 (13.9%)	125 (13.6%)	0.9708
Absent	323 (86.1%)	794 (86.4%)	
**TGFBR1 Mutation**			
Present	8 (2.1%)	13 (1.4%)	0.4928
Absent	367 (97.9%)	906 (98.6%)	
**TGFBR2 Mutation**			
Present	11 (2.9%)	28 (3.0%)	1
Absent	364 (97.1%)	891 (97.0%)	

Table S8. Comparison of Early-Onset Hispanic/Latino (H/L) versus Early-Onset Non-Hispanic White (NHW) Patients Treated with FOLFOX

TGF-beta Pathway			
Gene	Early-Onset Hispanic/Latino Treated with FOLFOX n (%)	Early-Onset NHW Treated with FOLFOX n (%)	p-value
**BMPR1A Mutation**			
Present	4 (5.5%)	4 (1.1%)	**0.02715**
Absent	69 (94.5%)	371 (98.9%)	
**SMAD2 Mutation**			
Present	4 (5.5%)	19 (5.1%)	0.7777
Absent	69 (94.5%)	356 (94.9%)	
**SMAD3 Mutation**			
Present	3 (4.1%)	14 (3.7%)	0.7469
Absent	70 (95.9%)	361 (96.3%)	
**SMAD4 Mutation**			
Present	9 (12.3%)	52 (13.9%)	0.8697
Absent	64 (87.7%)	323 (86.1%)	
**TGFBR1 Mutation**			
Present	2 (2.7%)	8 (2.1%)	0.6698
Absent	71 (97.3%)	367 (97.9%)	
**TGFBR2 Mutation**			
Present	3 (4.1%)	11 (2.9%)	0.7102
Absent	70 (95.9%)	364 (97.1%)	

Table S9. Comparison of Early-Onset Hispanic/Latino (H/L) versus Early-Onset Non-Hispanic White (NHW) Patients Not Treated with FOLFOX

TGF-beta Pathway			
**Gene**	**Early-Onset Hispanic/Latino Not Treated with FOLFOX n (%)**	**Early-Onset NHW Not Treated with FOLFOX n (%)**	**p-value**
**BMPR1A Mutation**			
Present	2 (3.8%)	10 (3.3%)	0.6916
Absent	50 (96.2%)	292 (96.7%)	
**SMAD2 Mutation**			
Present	3 (5.8%)	16 (5.3%)	0.7489
Absent	49 (94.2%)	286 (94.7%)	
**SMAD3 Mutation**			
Present	3 (5.8%)	12 (4.0%)	0.4701
Absent	49 (94.2%)	290 (96.0%)	
**SMAD4 Mutation**			
Present	7 (13.5%)	37 (12.3%)	0.9867
Absent	45 (86.5%)	265 (87.7%)	
**TGFBR1 Mutation**			
Present	2 (3.8%)	9 (3.0%)	0.668
Absent	50 (96.2%)	293 (97.0%)	
**TGFBR2 Mutation**			
Present	6 (11.5%)	16 (5.3%)	0.1583
Absent	46 (88.5%)	286 (94.7%)	

Table S10. Comparison of Late-Onset Hispanic/Latino (H/L) versus Late-Onset Non-Hispanic White (NHW) Patients Treated with FOLFOX

TGF-beta Pathway			
Gene	Late-Onset Hispanic/Latino Treated with FOLFOX n (%)	Late-Onset NHW Treated with FOLFOX n (%)	p-value
**BMPR1A Mutation**			
Present	0 (0.0%)	15 (1.6%)	0.386
Absent	91 (100.0%)	904 (98.4%)	
**SMAD2 Mutation**			
Present	3 (3.3%)	35 (3.8%)	1
Absent	88 (96.7%)	884 (96.2%)	
**SMAD3 Mutation**			
Present	4 (4.4%)	29 (3.2%)	0.5303
Absent	87 (95.6%)	890 (96.8%)	
**SMAD4 Mutation**			
Present	16 (17.6%)	125 (13.6%)	0.3753
Absent	75 (82.4%)	794 (86.4%)	
**TGFBR1 Mutation**			
Present	2 (2.2%)	13 (1.4%)	0.6379
Absent	89 (97.8%)	906 (98.6%)	
**TGFBR2 Mutation**			
Present	5 (5.5%)	38 (4.1%)	0.7334
Absent	86 (94.5%)	881 (95.9%)	

Table S11. Comparison of Late-Onset Hispanic/Latino (H/L) versus Late-Onset Non-Hispanic White (NHW) Patients Not Treated with FOLFOX

TGF-beta Pathway			
**Gene**	**Late-Onset Hispanic/Latino Not Treated with FOLFOX n (%)**	**Late-Onset NHW Not Treated with FOLFOX n (%)**	**p-value**
**BMPR1A Mutation**			
Present	0 (0.0%)	15 (2.3%)	0.6164
Absent	50 (100.0%)	638 (97.7%)	
**SMAD2 Mutation**			
Present	4 (8.0%)	43 (6.6%)	0.7661
Absent	46 (92.0%)	610 (93.4%)	
**SMAD3 Mutation**			
Present	4 (8.0%)	34 (5.2%)	0.3375
Absent	46 (92.0%)	619 (94.8%)	
**SMAD4 Mutation**			
Present	9 (18.0%)	104 (15.9%)	0.8532
Absent	41 (82.0%)	549 (84.1%)	
**TGFBR1 Mutation**			
Present	1 (2.0%)	12 (1.8%)	1
Absent	49 (98.0%)	641 (98.2%)	
**TGFBR2 Mutation**			
Present	3 (6.0%)	46 (7.0%)	1
Absent	47 (94.0%)	607 (93.0%)	

Table S12. Distribution of TGF-β Pathway Gene Mutation Types by Ancestry, Age of Onset, and FOLFOX Treatment Status in Colorectal Cancer. This table presents the percentage distribution of mutation types across six TGF-β pathway genes (BMPR1A, SMAD2, SMAD3, SMAD4, TGFBR1, and TGFBR2) stratified by ancestry [Hispanic/Latino (H/L) vs. Non-Hispanic White (NHW)], age of onset [early-onset (EO) vs. late-onset (LO)], and FOLFOX treatment status (treated vs. not treated). Mutation categories include frame shift deletions and insertions, in-frame deletions and insertions, missense mutations, nonsense mutations, nonstop mutations, splice site and splice region alterations, and translation start site variants. Percentages indicate the proportion of each mutation type among all mutations detected for a given gene within each subgroup. This table highlights differences in the mutation spectrum between clinical and demographic subgroups, enabling comparisons of variant type prevalence across ancestry, age, and treatment groups.

	Hispanic/Latino Samples				Non-Hispanic White Samples			
	Early-Onset		Late-Onset		Early-Onset		Late-Onset	
	Treated with FOLFOX	Not Treated with FOLFOX	Treated with FOLFOX	Not Treated with FOLFOX	Treated with FOLFOX	Not Treated with FOLFOX	Treated with FOLFOX	Not Treated with FOLFOX
**BMPR1A**								
Frame Shift Deletion	0.0%	0.0%	0.0%	0.0%	0.0%	0.0%	16.7%	15.8%
Frame Shift Insertion	0.0%	0.0%	0.0%	0.0%	25.0%	0.0%	5.6%	15.8%
In Frame Deletion	0.0%	0.0%	0.0%	0.0%	0.0%	0.0%	0.0%	10.5%
Missense Mutation	75.0%	100.0%	0.0%	0.0%	50.0%	45.5%	50.0%	42.1%
Nonsense Mutation	0.0%	0.0%	0.0%	0.0%	25.0%	45.5%	27.8%	10.5%
Splice Site	25.0%	0.0%	0.0%	0.0%	0.0%	9.1%	0.0%	0.0%
Translation Start Site	0.0%	0.0%	0.0%	0.0%	0.0%	0.0%	0.0%	5.3%
**SMAD2**								
Frame Shift Deletion	0.0%	0.0%	33.3%	0.0%	13.6%	4.8%	10.5%	13.3%
Frame Shift Insertion	0.0%	0.0%	0.0%	0.0%	4.5%	9.5%	10.5%	4.4%
Missense Mutation	60.0%	75.0%	66.7%	100.0%	50.0%	52.4%	50.0%	60.0%
Nonsense Mutation	40.0%	25.0%	0.0%	0.0%	27.3%	23.8%	28.9%	17.8%
Nonstop Mutation	0.0%	0.0%	0.0%	0.0%	4.5%	4.8%	0.0%	2.2%
Splice Site	0.0%	0.0%	0.0%	0.0%	0.0%	4.8%	0.0%	2.2%
**SMAD3**								
Frame Shift Deletion	0.0%	0.0%	0.0%	0.0%	0.0%	16.7%	3.0%	2.6%
Frame Shift Insertion	0.0%	0.0%	0.0%	20.0%	6.3%	5.6%	0.0%	13.2%
In Frame Deletion	0.0%	0.0%	0.0%	0.0%	0.0%	0.0%	3.0%	0.0%
Missense Mutation	100.0%	100.0%	75.0%	60.0%	75.0%	61.1%	69.7%	65.8%
Nonsense Mutation	0.0%	0.0%	25.0%	20.0%	18.8%	16.7%	18.2%	13.2%
Nonstop Mutation	0.0%	0.0%	0.0%	0.0%	0.0%	0.0%	3.0%	0.0%
Splice Site	0.0%	0.0%	0.0%	0.0%	0.0%	0.0%	3.0%	5.3%
**SMAD4**								
Frame Shift Deletion	0.0%	28.6%	21.1%	11.1%	3.4%	4.9%	11.1%	10.7%
Frame Shift Insertion	9.1%	14.3%	0.0%	0.0%	1.7%	4.9%	2.2%	2.5%
In Frame Deletion	0.0%	0.0%	0.0%	0.0%	1.7%	2.4%	0.0%	0.8%
In Frame Insertion	0.0%	0.0%	0.0%	0.0%	0.0%	2.4%	1.5%	0.0%
Missense Mutation	63.6%	57.1%	73.7%	77.8%	79.3%	70.7%	70.4%	68.0%
Nonsense Mutation	27.3%	0.0%	5.3%	0.0%	12.1%	14.6%	11.1%	13.1%
Nonstop Mutation	0.0%	0.0%	0.0%	0.0%	0.0%	0.0%	0.7%	0.0%
Splice Region	0.0%	0.0%	0.0%	0.0%	0.0%	0.0%	0.0%	0.8%
Splice Site	0.0%	0.0%	0.0%	11.1%	1.7%	0.0%	3.0%	3.3%
Translation Start Site	0.0%	0.0%	0.0%	0.0%	0.0%	0.0%	0.0%	0.8%
**TGFBR1**								
Frame Shift Deletion	0.0%	0.0%	0.0%	0.0%	10.0%	0.0%	0.0%	14.3%
In Frame Deletion	0.0%	0.0%	0.0%	0.0%	0.0%	0.0%	6.3%	7.1%
Missense Mutation	66.7%	100.0%	100.0%	100.0%	50.0%	81.8%	81.3%	71.4%
Nonsense Mutation	0.0%	0.0%	0.0%	0.0%	40.0%	18.2%	12.5%	7.1%
Translation Start Site	33.3%	0.0%	0.0%	0.0%	0.0%	0.0%	0.0%	0.0%
**TGFBR2**								
Frame Shift Deletion	0.0%	66.7%	40.0%	33.3%	27.3%	38.9%	23.3%	45.1%
Frame Shift Insertion	0.0%	0.0%	0.0%	0.0%	0.0%	5.6%	4.7%	3.9%
In Frame Deletion	0.0%	0.0%	0.0%	0.0%	0.0%	5.6%	2.3%	3.9%
In Frame Insertion	0.0%	0.0%	0.0%	0.0%	9.1%	0.0%	0.0%	0.0%
Missense Mutation	100.0%	33.3%	60.0%	66.7%	63.6%	50.0%	60.5%	39.2%
Nonsense Mutation	0.0%	0.0%	0.0%	0.0%	0.0%	0.0%	4.7%	3.9%
Splice Site	0.0%	0.0%	0.0%	0.0%	0.0%	0.0%	4.7%	3.9%

(a)

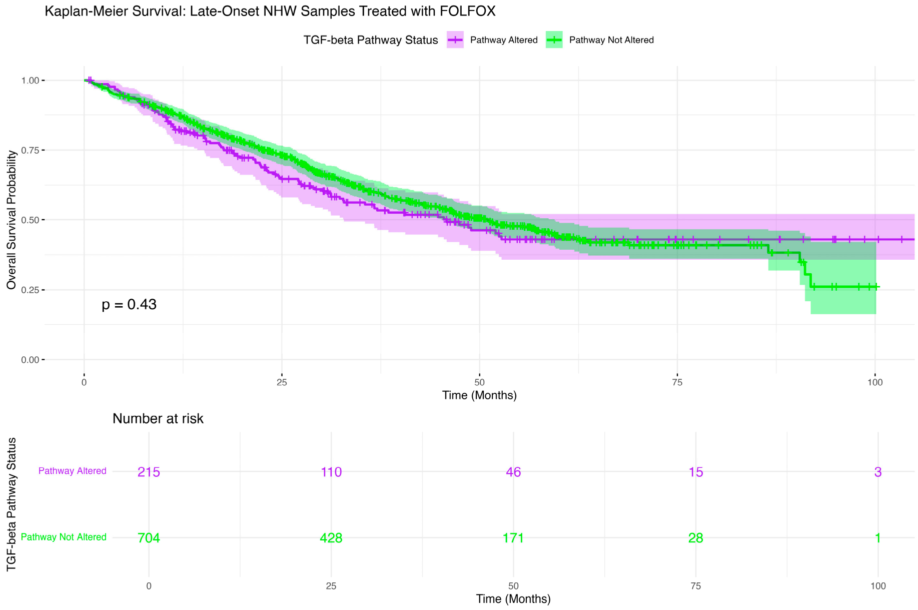

(b)

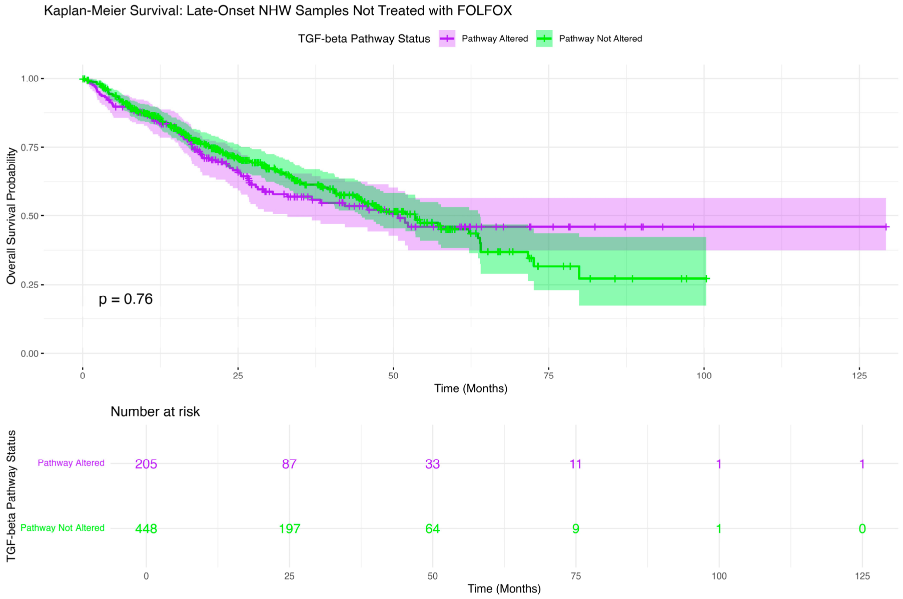

Figure S1. Kaplan–Meier survival analysis of TGF-beta pathway alterations across colorectal cancer (CRC) subgroups defined by age, ancestry, and FOLFOX treatment status. Overall survival curves are shown for: (a) Late-Onset NHW Treated with FOLFOX, and (b) Late-Onset NHW Not Treated with FOLFOX.

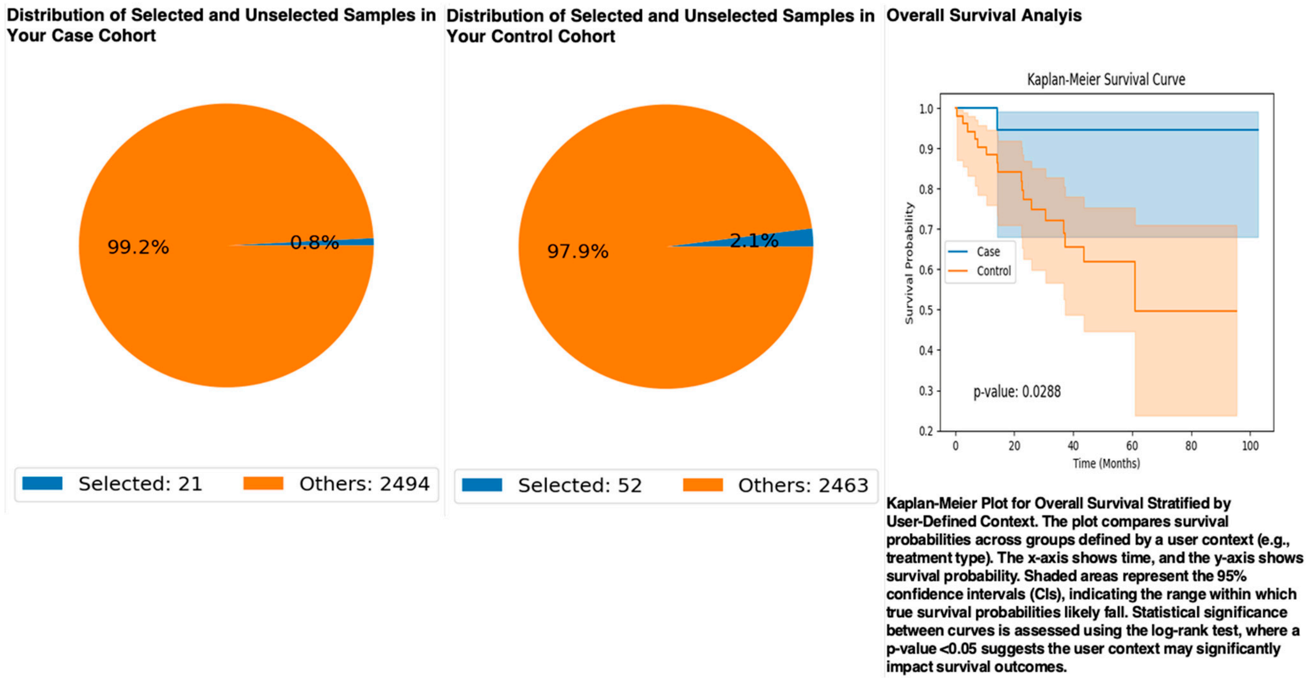

Figure S2. AI-guided selection and survival analysis of Hispanic/Latino (H/L) colorectal cancer (CRC) patients treated with FOLFOX, stratified by TGF-β pathway alteration status. The AI-HOPE and AI-HOPE-TGFbeta platforms were used to identify case and control cohorts based on predefined clinical, genomic, and treatment criteria. (Left) Distribution of selected versus unselected samples in the case cohort—early-onset (EO) H/L CRC patients treated with FOLFOX and harboring TGF-β pathway alterations (n = 21)—and the control cohort—late-onset (LO) H/L CRC patients treated with FOLFOX without pathway alterations (n = 52). (Right) Kaplan–Meier overall survival (OS) analysis comparing the two cohorts. Patients in the case group exhibited significantly reduced OS compared to controls (log-rank p = 0.029). Shaded areas represent 95% confidence intervals. Survival probabilities declined more steeply in the case cohort within the first 50 months, suggesting a potential negative prognostic impact of TGF-β pathway alterations in EO H/L patients receiving FOLFOX.

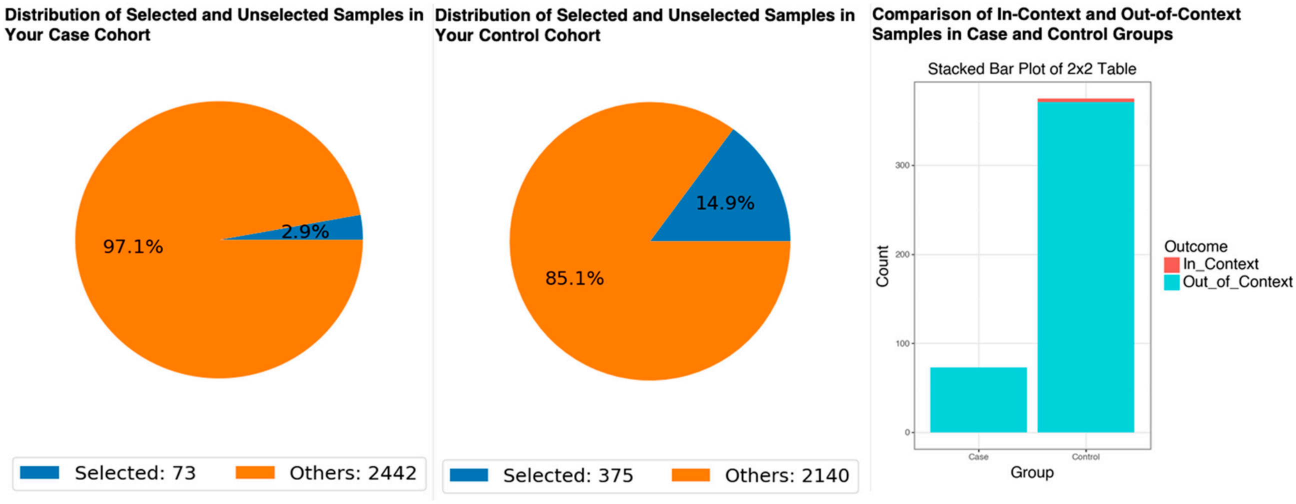

Figure S3. Comparison of BMPR1A mutation frequency between early-onset Hispanic/Latino (H/L) and early-onset Non-Hispanic White (NHW) colorectal cancer (CRC) patients treated with FOLFOX in the context of SMAD4 mutation positivity. The AI-HOPE and AI-HOPE-TGFbeta platforms were used to identify and compare BMPR1A mutation prevalence between the case cohort (EO H/L; n = 73) and control cohort (EO NHW; n = 375), restricted to patients harboring SMAD4 mutations. Pie charts show the proportion of selected (in-context) versus unselected (out-of-context) samples within each cohort. The stacked bar plot displays the number of in-context and out-of-context samples across case and control groups, with Fisher's exact test used to assess statistical significance. BMPR1A mutations were observed in 0.68% of cases and 1.07% of controls (p = 0.836; odds ratio = 0.0; 95% CI: 0.033–12.145), indicating no significant difference in mutation frequency between groups.

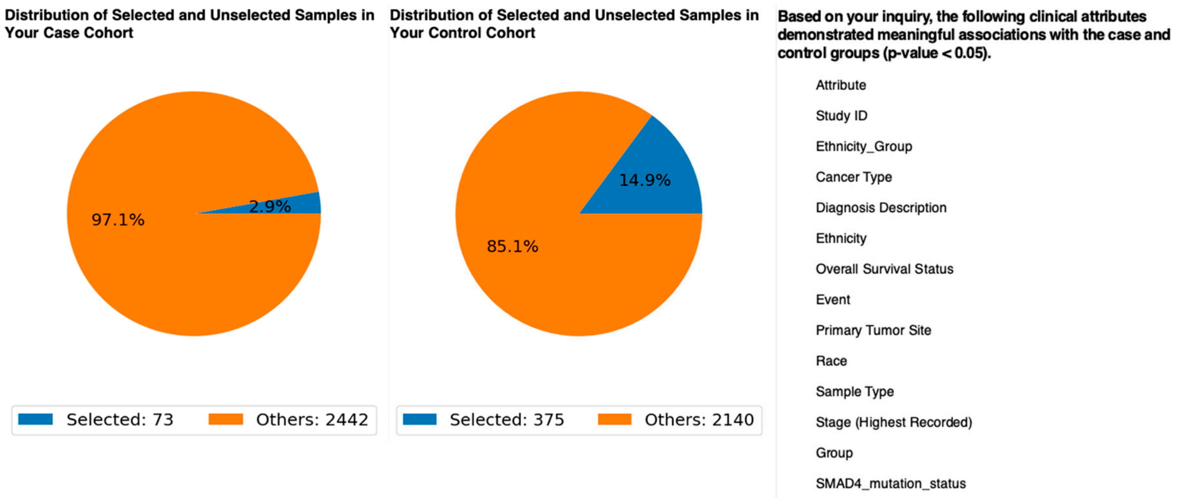

Figure S4. AI-driven identification of significant clinical and molecular attributes distinguishing early-onset Hispanic/Latino (H/L) and Non-Hispanic White (NHW) colorectal cancer (CRC) patients treated with FOLFOX. The pie charts display the proportion of selected versus unselected samples for the case cohort (early-onset H/L, n = 73) and control cohort (early-onset NHW, n = 375). The table lists all clinical and genomic attributes showing statistically significant differences between groups ($p < 0.05$), including demographic factors (ethnicity group, race), clinical variables (primary tumor site, stage, sample type), disease outcomes (overall survival status, event), and key oncogenic mutations (e.g., *SMAD4*). These results highlight multiple dimensions of divergence between cohorts, supporting further targeted analyses of ancestry-related differences in CRC biology and outcomes.

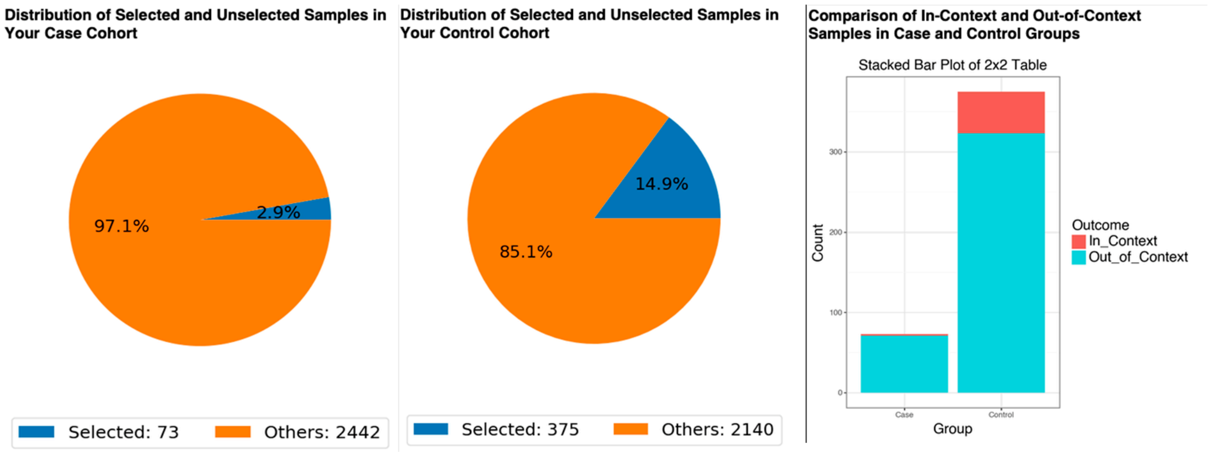

Figure S5. Comparison of *SMAD4* mutation prevalence between early-onset Hispanic/Latino (H/L) and Non-Hispanic White (NHW) colorectal cancer (CRC) patients treated with FOLFOX. The case cohort (left pie chart) consisted of early-onset H/L patients (n = 73) and the control cohort (middle pie chart) consisted of early-onset NHW patients (n = 375). Blue segments indicate selected samples meeting the inclusion criteria, while orange segments represent all other samples. The stacked bar plot (right) displays the distribution of *SMAD4*mutation–positive (“In_Context”) and mutation–negative (“Out_of_Context”) cases in each cohort. *SMAD4* mutations were detected in 2.74% of the case group compared with 13.87% of the control group. Fisher’s exact test yielded a statistically significant difference (Chi-square p = 0.013), with an odds ratio of 0.175 (95% CI: 0.042–0.735), indicating a lower prevalence of *SMAD4* mutations in early-onset H/L patients treated with FOLFOX relative to NHW counterparts.
